# Mechanistic insights into the pleiotropic effects of butyrate as a potential therapeutic agent on NAFLD management: A systematic review

**DOI:** 10.3389/fnut.2022.1037696

**Published:** 2022-12-02

**Authors:** Parichehr Amiri, Sara Arefhosseini, Farnush Bakhshimoghaddam, Hannah Jamshidi Gurvan, Seyed Ahmad Hosseini

**Affiliations:** ^1^Student Research Committee, Ahvaz Jundishapur University of Medical Sciences, Ahvaz, Iran; ^2^Nutrition and Metabolic Diseases Research Center, Clinical Research Institute, Ahvaz Jundishapur University of Medical Sciences, Ahvaz, Iran; ^3^Department of Nutrition, School of Allied Medical Sciences, Ahvaz Jundishapur University of Medical Sciences, Ahvaz, Iran; ^4^Student Research Committee, Tabriz University of Medical Sciences, Tabriz, Iran; ^5^National Medical Emergency Organization, Ministry of Health and Medical Education, Tehran, Iran

**Keywords:** NAFLD, butyrate, obesity, gut microbiota, insulin resistance

## Abstract

Non-alcoholic fatty liver disease (NAFLD) is one of the most common chronic diseases worldwide. As a multifaceted disease, NAFLD’s pathogenesis is not entirely understood, but recent evidence reveals that gut microbiota plays a significant role in its progression. Butyrate, a gut microbiota metabolite, has been reported to have hepato-protective effects in NAFLD animal models. The purpose of this systematic review is to determine how butyrate affects the risk factors for NAFLD. Searches were conducted using relevant keywords in electronic databases up to March 2022. According to the evidence presented in this study, butyrate contributes to a wide variety of biological processes in the gut–liver axis. Its beneficial properties include improving intestinal homeostasis and liver health as well as anti-inflammatory, metabolism regulatory and anti-oxidative effects. These effects may be attributed to butyrate’s ability to regulate gene expression as an epigenetic modulator and trigger cellular responses as a signalling molecule. However, the exact underlying mechanisms remain unclear. Human trials have not been performed on the effect of butyrate on NAFLD, so there are concerns about whether the results of animal studies can be translated to humans. This review summarises the current knowledge about the properties of butyrate, particularly its potential effects and mechanisms on liver health and NAFLD management.

## Introduction

Non-alcoholic fatty liver disease (NAFLD), recently renamed metabolic-associated fatty liver disease (MAFLD), has become the main cause of chronic liver disease (1, 2). NAFLD prevalence is increasing, as it affects almost one-quarter of the general population (3), and the number of NAFLD patients worldwide is estimated to reach 56% in the next 10 years (4). NAFLD includes a broad spectrum of liver conditions, from simple steatosis to non-alcoholic steatohepatitis (NASH), cirrhosis and even hepatocellular carcinoma (5). Moreover, as a multisystem disease, NAFLD is linked to other chronic morbidities such as diabetes and cardiovascular and chronic kidney diseases (5). The pathogenesis of NAFLD is based on a “multi-hit model” with parallel or synergic roles of genetic and epigenetic factors, including nutritional status, lifestyle, insulin resistance (IR), inflammation, oxidative stress, and altered gut–liver axis (GLA) (6).

Increased *de novo* lipogenesis and lipolysis in adipose tissue accompanied by reduced fatty acid oxidation in the liver results in various changes, including hepatic triglyceride (TG) accumulation, hypertriglyceridemia, hyperglycaemia, and eventually IR (7, 8). Increased hepatic fat deposits may activate protein kinase C, thereby inactivating insulin receptors and attenuating insulin sensitivity, leading to IR in the liver (9). IR is the foremost pathophysiological step in NAFLD progression, and it links NAFLD to metabolic dysfunction bidirectionally (10). Excessive hepatic TG deposits may also lead to lipotoxicity, mitochondrial dysfunction, radical oxygen species (ROS) generation, inflammation and DNA damage, which accelerates disease progression (8, 11, 12). Moreover, changes in pro- and anti-oxidant balance by decreasing the activity of glutathione (GSH) peroxidase and manganese dismutase may be observed during NAFLD pathogenesis (13). Lipotoxicity caused by contributing GLA, adipose tissue–liver axis and extracellular vesicles stimulates the activation of immune-inflammatory pathways *via* chemokines and cytokines (14). Evidence indicates that GLA dysfunction (ranging from bacterial overgrowth to gut microbiota dysbiosis) plays a pivotal role in NAFLD progression (15), and NAFLD patients exhibit significant GLA dysfunction compared to healthy subjects (16). Underlying mechanisms of GLA dysfunction include the hyperactivity of liver immune cells and increased intestinal pro-inflammatory metabolites (17, 18). These mechanisms change the secretion of interleukins (ILs), tumour necrosis factor α (TNF-α), and C-reactive protein (CRP), which results in a liver inflammatory response that worsens NAFLD (19, 20).

To our knowledge, approved pharmacotherapy for NAFLD is not yet available, and lifestyle interventions are known as the first-line treatments (21). However, clinical trials reveal that NAFLD is responsive to medication (6). According to previous studies, patients with NAFLD are mainly obese or overweight; thus, reducing weight by about 10% of the initial weight during 6–12 months along with taking specific dietary supplements (including anti-oxidant and anti-inflammatory drugs, vitamins, nutraceuticals, and probiotics) appears to be efficient (22).

Sodium butyrate (NaB) supplementation has recently shown some improvements in NAFLD (23). Butyrate, a member of short-chain fatty acids (SCFAs), is produced *via* the anaerobic microbial fermentation of non-digestible carbohydrates and is also found in some foods such as milk and butter (23–25). Butyrate is considered a primary energy supply for colonocytes in mammals and humans (26). Several studies have shown the positive effects of NaB on obesity, diabetes, metabolic syndrome and cancer (27). On the one hand, numerous animal studies have recently demonstrated the protective effects of NaB supplementation on NAFLD. There is also strong evidence regarding the anti-inflammatory, anti-oxidant and immunomodulatory effects of NaB on NAFLD. On the other hand, NaB influences lipid metabolism, gut homeostasis and IR (23), and it mainly acts as a histone deacetylase (HDAC) inhibitor, binding to specific G-protein-coupled receptors (GPCRs), thereby exerting its beneficial effects (28, 29). Therefore, this study aims to review the therapeutic efficacy of NaB and summarise the underlying mechanisms of the impact of NaB supplementation on NAFLD progression.

## Methods

### Search strategy

Searches were conducted in electronic databases, such as Scopus, ProQuest, Embase, PubMed, and Google Scholar, using the following keywords: “sodium butyrate” or “butyric acid” or “butanoic acid” or “butyrate” or “NaB” or “SoB” and “non-alcoholic fatty liver disease” or “fatty liver” or “NAFLD,” “non-alcoholic steatohepatitis” or “NASH” or “dyslipidaemia” or “high-fat diet” or “obesity” or “impaired fasting glucose” or “insulin resistance” or “HOMA-IR” or “oxidative stress” or “inflammation.” We included all keywords relevant to our primary objectives about butyrate’s effect on NAFLD risk factors to reduce the risk of missing studies. Additionally, we searched several databases, ensuring that most of the studies published so far were included in this review. Our search was limited to English language studies published up to March 2022.

### Eligibility criteria

This systematic review included studies that met the following criteria: (a) English language publications; (b) clinical trials; (c) *in vivo* models; (d) *in vitro* studies. We excluded studies with insufficient data, observational studies or those using butyrate-producing bacteria as well as studies on liver diseases other than NAFLD-related conditions.

### Data extraction and quality assessment of previous studies

The scientific literature was retrieved independently by two investigators based on the inclusion criteria. Studies that failed to meet the predefined criteria were excluded from further review. The quality assessment and data extraction of eligible studies were performed using a checklist containing the study aims, research question and inclusion and exclusion criteria. Afterward, a third person assessed the study’s accuracy, precision and quality. Any disagreement regarding study eligibility and quality assessment was resolved through discussion and consensus.

### Findings

**FIGURE 1:**
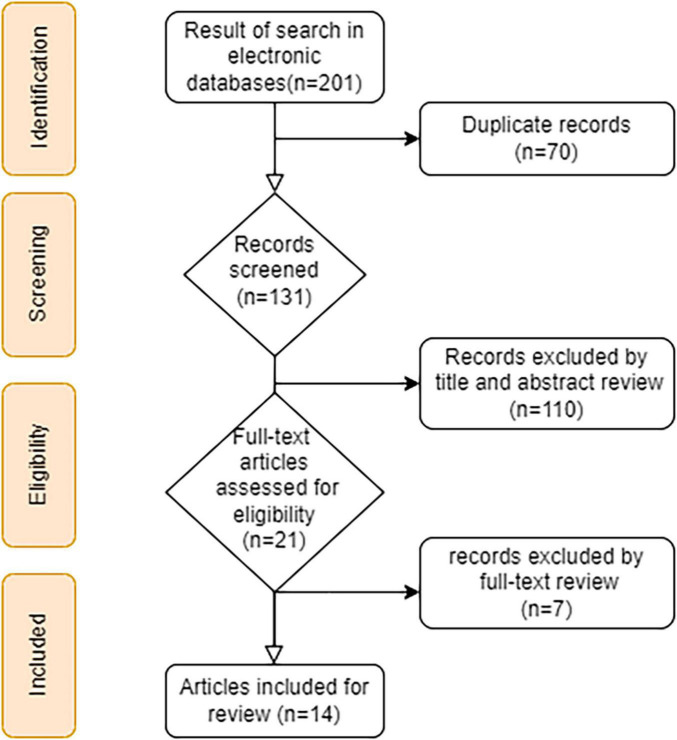
A flow chart showing the process of literature searching and selecting studies.

Figure 1 shows the process for selecting studies. The search strategy identified 201 relevant articles, and after duplicates were removed, the remaining 131 articles were screened. A further 110 articles were excluded as they failed to meet the inclusion criteria. In total, 21 full-text articles were reviewed, and when they were evaluated, seven studies were removed due to the exclusion criteria. The remaining 14 studies underwent qualitative syntheses. Table 1 shows 14 studies that evaluated the effects of NaB on NAFLD and associated risk factors. From each study, the following information was collected: first author’s name, year of publication, study location, type of animal/model, type of intervention, dose and duration of intervention, and effects of NaB on the gut, liver, and metabolic disorders, anti-inflammatory markers, and anti-oxidative status. Between 2013 and 2021, three studies were published in Germany (23, 30, 31), seven in China (25, 32–37), one in Netherlands (38), one in Austria (39), one in Italy (40), and one in Japan (41). Most studies used HFD-fed mice (32–34, 36, 37, 40), while the remaining used WSD (30, 31), FD (39), MCD (35), and FFC-fed (23) mice that supplemented with NaB. In included articles, the duration of interventions ranged from 12 h to 16 weeks, and NaB doses ranged from 20 mg/kg to 600 mg/kg. A total of nine studies reported the effects of NaB on expression of occludin (23, 25, 30, 31, 39), claudin-1 (35), claudin-2 (30), claudin-3 (30), claudin-5 (30), zonula occludens-1 (ZO-1) (23, 25, 30, 31, 35, 37, 39), glucagon-like peptide-1 (GLP-1) (25, 33, 36), TGR5 (25), lipopolysaccharide binding protein (LBP) (25, 35, 39), endotoxin (23, 37, 39), and dysbiosis (35, 37) were categorised as gut effect, 13 studies reported hepatic steatosis (25, 30–33, 35, 37, 38, 40), fibrosis (35, 38), hepatic TG (30, 33–35, 40), hepatic cholesterol (33, 34), fatty acid synthase (Fas) gene expression (41), expression of Carnitine palmitoyl transferase-1a (Cpt1a) gene (41), NAFLD activity score (NAS) (23, 32, 35, 37), alanine aminotransferase (ALT) (23, 25, 31–33, 35–37, 40, 41), aspartate aminotransferase (AST) (23, 25, 32, 33, 35–37, 40), and alkaline phosphatase (ALP) (25) were categorised as liver effects, 11 studies reported weight gain (23, 25, 30–32, 34, 36, 37, 39–41), liver/body weight ratio (23, 31, 39), and epididymal fat weight (34, 36, 37, 39) were categorised as obesity-induced NAFLD, four studies reported plasma TG (25, 30, 32, 40), total cholesterol (25, 32, 40), LDL-C (25, 32, 40), and HDL-C (32) were categorised as lipid metabolism disorders, five studies reported fasting blood glucose (FBG) (25, 32, 36, 37, 40), insulin (36, 37, 40), homeostatic model assessment of insulin resistance (HOMA-IR) (32, 36, 37), insulin sensitivity Index (ISI) (36, 37), and fasting serum insulin (FINS) (32) were categorised as glucose metabolism disorders, 13 studies reported IL-1 (37), IL-2 (35, 37), IL-3 (35), IL-4 (35, 37), IL-6 (23, 25, 30, 32, 34, 35, 37), IL-10 (35, 37), IL-12 (35), IL-17 (35), IL-1a (35), IL-1β (25, 30, 32, 34, 35, 40), TNFα (23, 25, 30, 32, 34–37, 39, 40), miR-150 (32), toll like receptor (TLR)-2 (35), TLR-4 (23, 39, 40), cpt1α (41), nuclear factor kappa-light-chain-enhancer of activated B cells (NF-kB) (31, 34, 40), TLR-4/Myd88/NF-kB (25, 37), histone deacetylase (HDAC) (34, 40), nucleotide-binding oligomerization domain-like receptor family pyrin domain-containing 3 (NLRP3) (34), M1 macrophage (34), peroxisome proliferator-activated receptors (PPARα) (34, 37), cluster of differentiation (CD)14 (35), monocyte chemo- attractant protein-1 (MCP-1) (37, 40), interferon (IFN) (37), and collagen cross-linking (CCL)-2 (39) were categorised as inflammation-induced NAFLD, and 13 studies reported uncoupling protein (UCP)-2 (38), superoxide Dismutase-1 (SOD1) (38), and FAS (31, 38), C-X-C chemokine receptor type 4 (CXCR4) (32), malondialdehyde (MDA) (32), superoxide Dismutase (SOD) (32), catalase (41), glutathione, 4-Hydroxynonenal (4-HNE) (23, 31, 39), inducible nitric oxide synthase (iNOS) (23, 31, 39, 40), melatonin (23, 39), Aco1 (41), SOD (41), Foxo3a (41), SOD (41), GSH (41), oxidised glutathione/glutathione ratio (GSSG/GSH) (41), cyclooxygenase (COX) (34, 40), plasminogen activator inhibitor-1 (PAI-1) (39), and high-mobility-group-box (HMGB-1) (40) were categorised as oxidative-induced NAFLD.

**TABLE 1:** Summary of the studies about the effects of NaB on NAFLD and associated risk factors.

Study	Animal/Model	Intervention/ Treatment	Dose and duration	Gut effects	Liver and metabolic effects	Anti-inflammatory and anti-oxidative effects
Beisner et al. Germany (30)	Female C57BL/6 mice	1. WSD + inulin 2. WSD + NaB 3. WSD 4. C	1. 10% inulin 2. 5% NaB 12 weeks	↑ expression of occludin, claudin-2, claudin-3, claudin-5, and ZO-1	↓hepatic lipid accumulation, ↓hepatic TG, ↓plasma TG, ↓weight gain	↓ IL-6 and TNFα in ileum, ↑ IL-1β in colon
Prins et al. Netherlands (38)	Precision-cut liver slices from Male C57/BL6 mice	1. C 2. NaB	1 mM NaB or Nacl 24 and 48 h of incubation	Not reported	↓hepatic steatosis, ↓fibrosis	↓UCP-2, SOD1, and FAS gene expression
Zhang et al. China (32)	Male C57BL/6J mice	1. C 2. HFD 3. HFD + MIM 4. HFD + NaB	1. MIM: 200 μL/day 2. NaB: 200 mg/kg/day 8 weeks	Not reported	↓hepatic lipid accumulation, ↓NAS, ↓total cholesterol, ↓TG, ↓LDL-C, ↑HDL-C, ↓AlT, ↓AST, ↓FBG, ↓HOMA-IR, ↔FINS, ↓weight gain	↓ IL-1β, IL-6, TNFα, CXCR4 and MDA gene expression ↑ miR-150 and SOD expression
Zhao et al. China (33)	Male C57BL/6 mice	1. HFD 2. HFD + NaB	200 mg/kg 8 weeks	↑GLP-1	↓liver steatosis, ↓hepatic TG, ↓hepatic cholesterol, ↓ALT, ↓AST	Not reported
Baumann et al. Germany (23)	Female C57BL/6J mice	1. C + NaB 2. FFC + NaB 3. C	600 mg/kg 8 weeks	↑occludin and ZO-1 proteins ↔endotoxin	↓NAS, ↓glucose tolerance, ↔ALT, ↔AST, ↓weight gain, ↓liver/body weight ratio	↓ TLR-4, IL-6, TNFα, 4-HNE gene expression, ↓iNOS protein, ↓ melatonin
Honma et al. Japan (41)	Sprague–Dawley male rats	1. C 2. NaB	5% NaB for 12 or 24 h	Not reported	↓Fas gene expression, ↑expression of Cpt1a gene, ↔weight gain	↑ Aco1, cpt1α, Mn-SOD, catalase, glutathione synthesis related genes, ↑SOD2 and Foxo3a gene expression, ↔ SOD, ↔GSH, ↔ GSSG/GSH
Yang et al. China (25)	Male C57BLKS/J background Lep^db^/Lep^db^ rats	1. C 2. C. butyricum 3. NaB	1. C. butyricum: 1.5 × 10^7^ CFU/kg 2. NaB: 500 mg/kg 6 weeks	↑GLP-1 through ↑TGR5 expression, ↓LBP ↑occludin and ZO-1 proteins	↓liver steatosis, ↓size of fat vacuoles, ↓total cholesterol, ↓TG, ↓LDL-C, ↓FBG, ↓ALT, ↓AST, ↓ALP, ↔ weight gain	↓ IL-1 β, IL-6, and TNFα gene expression ↓ TLR-4/Myd88/NF-kB protein levels
	HepG2 cells Caco-2 cells		NaB (2, 5, and 10 mM) 24 h			
Sun et al. China (34)	Male Sprague–Dawley rats	1. C 2. HFD 3. HFD + NaB	300 mg/kg 9 weeks	Not reported	↓hepatic TG, ↓hepatic cholesterol, ↓weight gain, ↓Epididymal fat weight	↓activation of HDAC, ↓NF-kB, NLRP3, TNFα IL-1β, and IL-6 gene expression, ↓ M1 macrophage F4/80, ↑M2/CD206, ↑PPARα, COX1, and COX4 protein, ↑activity of mitochondrial complex II and V and fatty acid β-oxidation
Ye et al. China (35)	Male C57BL/6 J mice	1. Control 2. MCD 3. C + NaB 4. MCD + NaB	600 mg/kg 6 weeks	↑claudin-1 and ZO-1 expression ↓LBP ↓dysbiosis	↓hepatic lipid accumulation, ↓NAS, ↓hepatic TG, ↓fibrosis, ↓ALT, ↓AST	↓activation of TLR2, ↓TLR4, ↓CD14, ↓IL-1a, ↓IL-1 β, ↓IL-2, ↓IL-3, ↓IL-6, ↓IL-12, ↓IL-17, ↓TNFα ↑IL-4, ↑IL-10
Zhou et al. China (36)	Male C57BL/6 mice	1. C 2. HFD 3. HFD + NaB	200 mg/kg 16 weeks	↑GLP-1 ↑GLP-1R expression	↓liver index, ↓FBG, ↔insulin, ↔HOMA-IR, ↔ISI, ↓ALT, ↓AST, ↓weight gain, ↔Epididymal fat weight	↔TNFα related to GLP-1
	HepG2 cells		NaB (1, 2, 5, 10 mM)			
Zhou et al. China (37)	Male C57BL/6 mice	1. C 2. HFD 3. HFD + NaB	200 mg/kg 8 weeks	↑ ZO-1 expression ↓endotoxin ↓dysbiosis	↓hepatic lipid accumulation, ↓NAS, ↓weight gain, ↓FBG, ↔insulin, ↓HOMA-IR, ↑ISI, ↓ALT, ↓AST, ↓weight gain, ↔Epididymal fat weight	↓MCP-1, TNFα, IL-1, IL-2, IL-6, IFN, and TLR-4/MyD88 gene expression, ↑ IL-4, IL-10 and PPARs gene expression, ↓ lipid peroxidation
Jin et al. Austria (39)	Male C57BL/6J mice	1. C 2. FD 3. FD + NaB	600 mg/kg 6 weeks	↑occludin and ZO-1 proteins ↔endotoxin levels, ↔expression of LBP in liver	↔weight gain, ↔liver/body weight ratio	↓TRL-4, CCL-2, TNFα, iNOS, 4-HNE gene expression, and PAI-1 protein levels, ↑duodenal melatonin and related enzymes synthesis
	Caco-2 cells J774A.1 cells					
Jin et al. Germany (31)	Female C57BL/6J mice	1. C 2. WSD 3. C + NAB 4. WSD + NaB	600 mg/kg 6 weeks	↑occludin and ZO-1 proteins	↓hepatic lipid accumulation, ↔FBG, ↔ALT, ↔weight gain, ↓liver/body weight ratio	↓NF-kB, iNOS, FAS, 4-HNE gene expression
Mattace Raso et al. Italy (40)	Male Sprague–Dawley rats	1. C 2. HFD 3. HFD + NaB 4. HFD + FBA	1. NaB: 20 mg/kg 2. FBA: 42.5 mg/Kg 6 weeks	Not reported	↓liver steatosis, ↓lipid accumulation, ↓hepatic TG content, ↓AST, ↓ALT, ↓total cholesterol, ↓LDL-c, ↔TG, ↔insulin, ↓FBG, ↔weight gain	↓activation of HDAC, TLRs, NF-kB, TNFα, MCP1, IL-1β, IL-6, HMGB-1, COX2, and iNOS gene expression, ↓liver inflammatory damage

In this table shows 14 studies that evaluated the effects of NaB on NAFLD and associated risk factors (↓decreased, ↑increased, ↔ not changed). Aco1, aconitase 1; ALT, alanine aminotransferase; AST, aspartate aminotransferase; C, control; Caco-2, carcinoma colon-2; COX, cyclooxygenase; CD, cluster of differentiation; Cpt1a, carnitine palmitoyl transferase 1a; CXCR4, C-X-C chemokine receptor type 4; Fas, fatty acid synthase; FBA, N-(1-carbamoyl-2-phenyl-ethyl) butyramide; FBG, fasting blood glucose; FD, fructose-enriched liquid diet; FFC, fat-, fructose- and cholesterol-rich diet; FINS, fasting serum insulin; GLP1, glucagon-like peptide-1; GLP-1R, GLP-1 receptor; GSH, glutathione; GSSG/GSH, oxidised glutathione/glutathione ratio; HDAC, histone deacetylase; HDL-c, high-density lipoprotein cholesterol; HFD, high-fat diet; HMGB, high-mobility-group-box; 4-HNE, 4-Hydroxynonenal; HOMA-IR, homeostatic model assessment of insulin resistance; IFN, interferon; IL, interleukin; iNOS, inducible nitric oxide synthase; ISI, insulin sensitivity Index; LBP, lipopolysaccharide binding protein; LDL-c, low-density lipoprotein cholesterol; MCD, methionine–choline-deficient diet; MCP1, monocyte chemo- attractant protein-1; MCS, methionine–choline-sufficient diet; MDA, malondialdehyde; MIM, metabolites of intestinal microflora; miR, microRNA; Mn-SOD, manganese superoxide dismutase; NaB, sodium butyrate; NAS, NAFLD activity score; NF-kB, nuclear factor kappa-light-chain-enhancer of activated B cells; Nrf2, nuclear respiratory factor-2; PAI-1, plasminogen activator inhibitor-1; PPAR, peroxisome proliferator-activated receptors; SOD1, superoxide Dismutase-1; TG, triglyceride; TLR, toll like receptor; TNFα, tumour necrosis factor α; UCP2, uncoupling protein-2; WSD, western-style diet; ZO-1, zonula occludens-1.

## Butyrate, a short-chain fatty acid derived from gut microbiota

### Fermentation and metabolization of butyrate

In the lumen of the intestine, butyrate is mainly produced by several anaerobe bacteria belonging to the Firmicutes and Bacteroidetes families (42). The primary sources fermented to butyrate are indigestible food components like carbohydrates (dietary fibre and resistant starch), although proteins can also be fermented to butyrate in smaller amounts (43). From carbohydrates, butyrate is synthesised *via* glycolysis when combining two acetyl-CoA to produce acetoacetyl-CoA, a stepwise reduction to butyryl-CoA (43, 44). Then, butyrate is formed from butyryl-CoA in two pathways: (1) *via* acetate CoA-transferase converted to butyrate and acetyl-CoA; (2) *via* phosphotransbutyrylase converted to butyryl phosphate, which forms butyrate *via* butyrate kinase (43, 44). Butyrate can also be produced *via* the lysine pathway from proteins (43). In the colon, butyrate is absorbed and partially metabolised by the colonocytes, and the remainder enters the liver *via* the portal vein (40). The colonocytes uptake butyrate *via* different exchange methods and transporters (40, 43). A high hepatic clearance leads to small amounts of butyrate reaching systemic circulation (45). However, even small amounts of butyrate exert a plethora of effects.

## Mechanisms of butyrate, a pleiotropic metabolite in NAFLD management

The main underlying mechanisms of butyrate’s beneficial effects are (a) its epigenetic acting as an HDAC inhibitor that affects several gene expressions in different pathways in the body; (b) its ability to bind to numerous specific GPCRs, which triggers and initiates intracellular responses (28, 29). As well as the emerging impacts of butyrate on the intestinal tract, like improving gut health and barrier function, many studies have found extra-intestinal effects *via* the GLA and gut–brain axis (43). The literature indicates that butyrate can cross the blood–brain barrier through mono-carboxylate transporters located on endothelial cells, and its concentrations are about an order of magnitude higher in wet brain samples than in peripheral blood (24, 46). Several mechanisms of butyrate’s actions in NAFLD management are discussed in the next three sections, including the effects of butyrate on the gut, liver and risk factors (obesity, dyslipidaemia, glucose dysmetabolism, inflammation, oxidative stress).

### The effects of butyrate on the gut

Non-alcoholic fatty liver disease development and progression may be influenced by overnutrition, genetic predisposition and changes in gut microbiota and intestinal barrier functions, which may develop into elevated endotoxin levels and increased permeation of bacterial endotoxins (39). Research has also demonstrated that gut microbiota and bacteria metabolites play an essential role in regulating the body’s metabolic processes (18). On the one hand, it has recently been suggested that butyrate, as an active bacterial metabolite, can help maintain gastrointestinal (GI) homeostasis (31, 47). On the other hand, butyrate-producing bacteria in healthy subjects is higher than in patients with NASH or NAFLD (48). Similarly, in animal models of NAFLD, butyrate concentrations were significantly lower in the faeces of mice fed with a high-fat diet (HFD) than in controls (49). It was also found that the serum and stool levels of butyrate in NAFLD patients were much lower than those in healthy subjects (50). Primarily, butyrate is essential for the nourishment of epithelial cells in the intestine (47). Moreover, it is thought that butyrate may help treat liver disease by affecting the GLA, including improving intestinal barrier function, regulating gut hormone secretion and inhibiting pathogenic bacteria growth (37).

Both *in vitro* and *in vivo* studies suggest that butyrate affects intestinal homeostasis by improving intestinal integrity and modulating tight junction (TJ) proteins (39, 51–53). TJs are multiprotein junctional complexes that are also named occluding junctions or zonulae occludes (54). The permeability of the epithelial barrier is maintained by TJs, which play a vital role in preventing harmful substances, such as bacteria and endotoxins, enter the bloodstream (51). A damaged TJ can cause chronic inflammation in various organs (25, 55), and several studies have reported the disruption of TJ integrity in NAFLD (25, 56). Jin et al. studied mice fed with a western-style diet (WSD) (31) and reported that oral supplementation with NaB (600 mg/kg) could restore the damaged intestinal mucosa and strengthen the TJs in the gut. Butyrate has been shown to restore TJ barrier function by activating zonula occludens-1 and occludin proteins, which are mediated by their ability to inhibit HDAC (31). These findings appear to be supported by several studies (23, 25, 35, 37, 39).

Treatment with butyrate may stabilise the disrupted TJs’ structure. Therefore, by improving gut permeability, NaB reduces the concentration of serum endotoxins and inflammatory cytokines, which are linked to liver diseases (39). Endotoxins, like lipopolysaccharide (LPS), are ligands for TLRs, so serum LBP levels are considered an indirect indicator of endotoxemia (35). Ye et al. found that NaB significantly reduced serum LBP levels and subsequent TLR2 and TLR4 messenger ribonucleic acid (mRNA) expressions (35). Likewise, Yang et al. showed that butyrate treatment *in vitro* and *in vivo* ameliorated the disruption of intestinal TJs in 16-week-old db/db mice as well as in high glucose-cultured carcinoma colon-2 (Caco-2) cells (25). Subsequently, NaB decreased inflammation in the intestine and LPS-treated Caco-2 cells and upregulated Takeda G-protein-coupled expression in the intestinal tissues by increasing serum GLP-1 levels (25).

Studies reported that NaB could induce the release of GLP-1 from entero-endocrine L cells (25, 33, 36). The GLP-1 hormone is key to the management of both diabetes type 2 (T2D) and obesity (36). GLP-1 regulates calorie intake, GI motility and glucose homeostasis (36), and research suggests that it may be a novel treatment for NASH, as it exerts direct and beneficial effects on hepatocytes, preventing their progression from NAFLD to NASH (57, 58).

Ye et al. revealed that microbiota dysbiosis caused by a methionine–choline-deficient diet was alleviated by butyrate and improved the metabolomic profile of faeces (35). Dysbiosis disrupts microbiota homeostasis caused by an imbalance in the microflora, changes in functional composition and metabolic activities or a shift in local distribution, which was revealed to be related to NAFLD onset and development (59). These findings show that butyrate supplementation might contribute to the amelioration of NAFLD by modifying gut microbiota and faecal metabolites, increasing intestinal integrity and modulating TJ proteins.

### The effects of butyrate on the liver

NAFLD encompasses a group of histopathological abnormalities, including benign steatosis, lobular inflammation and hepatic ballooning degeneration that may lead to liver fibrosis (10). Moreover, significant increases in serum ALT and AST levels, in the case of excluding other liver disorders, could manifest NAFLD’s incidence up to 90% (60). NaB supplementation in several pharmacological doses (200–600 mg/kg/day) has shown favourable hepato-protective effects. Different pathways, such as the modifying gut microflora, intestinal mucosal barrier and gut endotoxins-induced systemic inflammation, are involved in this manner (23, 30, 37, 39). NaB improves liver health by reducing hepatic lipid accumulation, liver TG and total cholesterol (TC) content, serum liver enzymes as well as alleviating liver fibrosis (35). Mattace-Raso et al. (40) reported significant improvements in liver damage, steatosis and a reduction in hepatic TG content after NaB administration, which is in agreement with many studies (30, 33–35, 39). The results of two studies by Jin et al. (31, 39) suggested that supplementation with NaB may protect HFD-fed mice from hepatic steatosis and NASH. Beisner et al. (30) demonstrated reduced liver weight gain and hepatic TG content in WSD-fed mice that received NaB for 12 weeks. More precisely, another study supplemented HFD-fed mice with 200 mg/kg/day NaB for 16 weeks, and intrahepatic TG and TC were approximately 0.33 times lower in the NaB-receiving group compared with controls (37). Despite the cumulative evidence confirming these positive changes, some studies report that liver features do not change significantly in NaB-fed animals (23). NaB’s producing strain, *Clostridium butyricum*, may also improve hepatic steatosis and decrease the size of fat vacuoles (25). Results have shown that NaB administration can ameliorate liver steatosis by reducing intrahepatic TG deposition. Its potential mechanisms highlight the importance of NaB in cellular metabolism through the main metabolic signalling pathways to modulate hepatocytes’ lipid metabolism. The underlying mechanisms of NaB’s action in this context may be classified into (1) inhibiting hepatic *de novo* lipogenesis through regulating involved genes; (2) increasing fatty acid oxidation *via* HDAC inhibition activity; (3) upregulating miRNAs involved in pathways related to lipid metabolism; (4) stimulating mitochondrial β-oxidation through hepatic peroxisome proliferator-activated receptor α (PPARα) activation.

Non-alcoholic fatty liver disease is characterised by parallel changes (i.e., fat deposition), followed by bidirectional changes in IR and hepatic *de novo* lipogenesis (10). Targeting IR-dependent lipogenesis could be a goal for NAFLD (61, 62). An insulin-induced gene (INSIG), as a potent inhibitor of sterol regulatory element-binding protein (SREBP) transcription factors, plays a critical role in *de novo* lipogenesis (63). Jin et al. (31) and Honma et al. (41) illustrated that NaB supplementation reduces the amount of fat infiltrated into liver cells and decreases predominant microvascular hepatic fat deposition. NaB significantly attenuates the induction of hepatic FAS mRNA expression without affecting the expression of SREBP-1, thereby reducing the liver’s TG content (31, 41). Zhao et al. (33) found that the administration of NaB caused a reinforcement of INSIG activity and suppressed *de novo* lipogenic genes, leading to enhanced phosphorylation of adenosine monophosphate-activated protein kinase (AMPK) in mice fed with an HFD diet. AMPK is an intra-cell metabolism regulator that may directly inhibit SREBP-1 activity, thereby preventing hepatic lipogenesis (64). Indeed, NaB increases the hepatic expression of the GLP-1 receptor by inhibiting HDAC2, which increases fatty acid oxidation and inhibits lipid synthesis in hepatocytes, thereby reducing hepatic steatosis (36).

Ex-4 is an analogue of GLP-1 that is widely used to regulate blood sugar through various organs such as the brain and pancreas (65). Ex-4 can effectively treat HFD-induced NAFLD by reducing hepatic steatosis with the end product of advanced glycation (65). Yang et al. (25) demonstrated that Ex-4 can reduce lipid accumulation in high glucose and free fatty acid co-cultured HepG2 cells, indicating the effectiveness of the GLP-1 analogue in managing NAFLD by regulating lipid metabolism in hepatocytes. NaB may also act through gene modifications. *In vivo* studies have illustrated the genetic mechanism of NaB action through the miR-150/chemokine receptor 4 (CXCR4) axis (32). miRNAs, which are small, non-coding and endogenous RNAs, are involved in regulating fatty acid and cholesterol metabolism and are among the novel therapeutic targets in NAFLD (66). miR-150 plays a vital role in the expression of genes associated with fatty acid uptake and the β-oxidation of fatty acids (32).

CXCL12 is expressed in the liver and is involved in several pathological disorders, for example, cancer and autoimmunity (67). Zhang et al. (32) found that 200 mg/kg/day of NaB might alleviate NAFLD by upregulating miR-150 expression to inhibit CXCR4 expression in HFD-fed mice, eventually relieving liver steatosis. NAFLD is also a mitochondrial disorder due to hepatic mitochondrial dysfunction during multi-hit pathogenesis. Hence, NaB targets hepatic mitochondria and reduces fat accumulation in liver disorders (68). Mattace-Raso et al. (40) reported the effects of NaB on the activation of PPAR-α. Hepatic PPARα activity can reverse NAFLD by stimulating mitochondrial β-oxidation (69).

Conversely, the hepatic-specific deletion of PPARα impairs fatty acid catabolism, resulting in hepatic lipid accumulation and NAFLD (69). It has been reported that NaB may restore the hepatic PPARα expression suppressed by an HFD, which suggests that PPARα could potentially mediate the butyrate function in alleviating NAFLD (40). Sun et al. (34) reported the effects of NaB intervention on a significant reduction in liver weight and lipid deposition through the significant upregulation in hepatic PPARα. Lowered hepatic TG without changes in hepatic TC concentrations was also reported in this study (34). An *ex vivo* study on a MAFLD model was conducted, and precision-cut liver slices were obtained to examine the direct effect of NaB on liver tissue (38). It was documented that NaB could improve the fibrotic response of the liver slices (38). Additionally, although it increased C4-related carnitines, which indicate butyrate oxidation, the expression of genes encoding fatty acid oxidation reduced (38). The data in this study demonstrated that NaB supplementation may be an efficient strategy for the prevention of MAFLD (38).

Serum liver enzymes are non-invasive, combinatory biomarkers used in assessing NAFLD (70). Yang et al. (25) investigated the hepato-protective effects of NaB and *Clostridium butyricum* on diabetes-induced NAFLD after 16 weeks. *Clostridium butyricum* prevented liver enlargement and decreased liver index and serum ALT, AST, and alkaline phosphatase concentrations (25). Another study revealed a significant reduction in the plasma concentration of ALT and AST after NaB administration (23). Most studies (25, 33, 35, 37, 40) reported the potential of NaB in reducing serum liver enzymes, while some studies did not show any significant changes (23, 31).

Based on GLA, the development of dietary approaches for modulating the intestinal environment seems effective to our knowledge. In this context, NaB is a beneficial strategy that improves the intestinal microbiome and subsequently ameliorates liver function. Briefly, the intestinal microbiome regulates glucose, lipid metabolism and metabolic homeostasis, thereby contributing to the progression of hepatic steatosis. Furthermore, NaB supplementation *via* the mechanisms mentioned above reduces hepatic lipid accumulation and prevents the development of NAFLD. Nevertheless, the exact signalling pathways are not fully understood, and future studies are needed to investigate the mechanisms and determine the effects of oral NaB supplementation in human clinical trials.

### The effects of butyrate on NAFLD risk factors

#### The effects of butyrate on obesity-induced NAFLD

A growing body of evidence suggests that butyrate is distributed beyond the gut to the central nervous system and peripheral tissues, including white and brown adipose tissue, which regulates whole-body energy metabolism, substrate metabolism and the development of NAFLD in animal models (71). The effects of butyrate under chow diet-fed conditions remain controversial. Previous studies indicated that butyrate could reduce appetite and food intake by stimulating the secretion of GI hormones, including GLP-1 and peptide YY (PYY), as anorexic hormones (72). Furthermore, several studies highlight butyrate’s role in modifying intestinal barrier integrity and the modulation of intestinal microbiota as a management strategy for regulating energy homeostasis (27, 73). Beisner et al. indicated that 5% NaB (mg/kg) supplementation reduced HFD-induced body weight gain in mice compared with HFD only (30). For eight consecutive weeks, rats treated with NaB (200 mg/kg/day) had a lower body weight gain (33). Four studies agreed that NaB reduced weight gain in HFD-fed mice (23, 34, 36, 37), although other studies showed no changes in body weight or body composition after NaB treatment (25, 31, 39–41). The anti-obesity potential of butyrate remains controversial and needs further investigation. The effects of butyrate in different doses, interventional periods and delivery methods should be validated to accurately determine the effects of butyrate on energy harvest and obesity.

#### Butyrate and lipid metabolism disorders

Dyslipidaemia appears to significantly influence the development and progression of the metabolic disorders associated with NAFLD (74). Butyrate can potentially regulate lipoprotein metabolism in the liver and gut, and some evidence has confirmed that it exerts favourable effects on liver disorders (75). The effect of butyrate administration on lipid metabolism in mice with NAFLD was assessed in four studies (25, 30, 32, 40). Yang et al. (25) observed that NaB (500 mg/kg NaB per day for 6 weeks) restored the elevated serum TG, TC and low-density lipoprotein cholesterol (LDL-C) concentrations in 16-week-old T2D-induced NAFLD mice. Beisner et al. (30) fed mice with a WSD, and they obtained higher plasma TG levels, which were significantly reduced when NaB (5% mg/kg for 12 weeks) was supplemented. In the study mentioned above, the lipid profile improved due to weight loss and decreased hepatic fat accumulation due to NaB. Additionally, Zhang et al. (32) and Mattace-Raso et al. (40) demonstrated that intervention with NaB decreased the serum contents of TC, TG, LDL-C and high-density lipoprotein cholesterol in the NAFLD mouse model. From the results of these studies, it can be concluded that butyrate, as a regulator, is involved in improving the lipid profile by inducing fatty acid oxidation and reducing lipogenesis (76).

Butyrate can attenuate hypercholesterolemia by downregulating the expression of crucial genes for the biosynthesis pathway of cholesterol in the intestine (77). Cholesterol homeostasis is achieved by closely regulating dietary absorption, biosynthesis, esterification and excretion. In the liver and intestine, cholesterol is esterified or released as a prime component of plasma lipoproteins, including chylomicrons, very-low-density lipoprotein (VLDL), LDLs and high-density lipoprotein (78). In general, butyrate’s inhibitory effect was seen in apolipoprotein B-48 output, TG export and chylomicron and VLDL secretion (79). Moreover, butyrate significantly reduced cholesterol synthesis by decreasing 3-hydroxy-3-methylglutaryl coenzyme A reductase (HMG-CoA reductase) levels in the liver microsomes of rats (80). It is suggested that the reverse cholesterol transport system played a significant role in atherosclerosis progression, stimulating the cholesterol movement from the peripheral tissues to the liver for re-use or excretion into the bile acid and by upregulating cytochrome P450 7A1 (81). Butyrate promotes reverse cholesterol transport and activates adenosine triphosphate-binding cassette transporter genes that stimulate cholesterol efflux from cells to lipid-free apolipoprotein A1 and transport them to the liver for further metabolism (82). Based on these mechanisms, butyrate can be proposed as a novel management strategy to improve lipid and lipoprotein homeostasis.

#### Butyrate and glucose metabolism disorders

It is known that NAFLD is an integral part of the metabolic syndrome, which comprises dysglycaemia and IR as central pathogenic factors (83). The comorbidities and complications of impaired glucose homeostasis correlate with increased opportunistic pathogen load and declined butyrate-producing bacteria levels in the intestine (59). Two animal studies have demonstrated the beneficial effects of NaB supplementation on glucose tolerance and fasting blood sugar (FBS) (23, 25). Hyperglycaemia can cause advanced IR and a responding failure of functional pancreatic beta cells (islet failure) to preserve appropriate insulin output and compensate for decreased insulin sensitivity (84). G-protein-coupled receptor (GPR) 41 and GPR43 are two primary receptors influenced by SCFAs, especially butyrate expressed by beta cells (85). Some evidence has stated that butyrate can act as ligands of GPR41 and GPR43, which induce the secretion of the GLP-1 and play a role in improving diabetes-induced histological alteration of islet and functional damage (86). Evidence suggests that the anti-diabetic effects of butyrate are related to its function as an HDAC inhibitor, which modifies hyperglycaemia by controlling the glucose-6 phosphate expression and the subsequent gluconeogenesis (87, 88). Furthermore, butyrate enhances the release of GLP-1, which plays a crucial role in the regeneration of beta cells and is referred to as a differentiation-inducing agent for insulin-producing cells (89). GLP-1 activates the GLP-1 receptor (GLP-1R) in pancreatic beta cells, resulting in and promoting glucose-stimulated insulin secretion (90).

Zhou et al. found that serum GLP-1 concentrations were significantly elevated after NaB treatment, although improved insulin sensitivity did not reach statistical significance in HFD-fed NAFLD mice (36). Recently, Zhang et al. administered 200 mg/kg/day of butyrate in HFD-induced NAFLD mice and found that it was negatively associated with FBS and IR; however, the intervention with butyrate failed to alter insulin concentrations (32). The effects of butyrate on insulin levels were consistent in different studies. Mattace-Raso et al. and Zhou et al. reported that butyrate supplementation significantly decreased homeostatic model assessment for IR (HOMA-IR) and FBS levels without changes in insulin concentration (37, 40). However, Jin et al. found that FBS concentrations did not differ after NaB intervention (600 mg/kg) in a NASH animal model, although insulin receptors were markedly higher in the livers of the NaB-supplemented group (31). The study’s results suggest that butyrate exerts its effects more by increasing insulin sensitivity to glucose metabolism. Overall, the studies mentioned earlier suggest that butyrate may protect mice from HFD-induced glucose metabolism disorders. The activation of GLP-1R and GPRs and the inhibition of HDAC may contribute to glucose homeostasis regulation; therefore, butyrate and its derivatives may have potential applications in preventing and managing metabolic disorders.

#### The effects of butyrate on inflammation-induced NAFLD

Growing evidence suggests that inflammation is a critical precursor of NAFLD (91). Excessive hepatic fat deposits trigger the impairment of an inflammatory response (92). Moreover, the gut microbiota system and its metabolites can change the immuno-inflammatory state (19, 93), which is indicated by the abnormal production of chemokines, cytokines and inflammatory markers (94). An ascending trend in serum pro-inflammatory mediators like ILs, NF-kB, TNF-α and other general markers have previously been shown in NAFLD (95). Previous findings propose managing the inflammatory response as an optimal target in treating NAFLD. As mentioned above, therapeutic pharmacological doses of NaB (200–600 mg/kg/day) have demonstrated many beneficial properties, including anti-inflammatory responses in different tissues (23, 96, 97).

Mattace-Raso et al. (40) first demonstrated that the administration of NaB and its synthetic derivative, phenylalanine butyramide (20 and 42.5 mg/kg/day, respectively), for 6 weeks significantly reduced inflammation in HFD-fed rats with steatosis by suppressing the NF-kB pathway. In this line, subsequent studies also reported the inhibition of the NF-kB pathway after NaB supplementation (25, 31, 34). One of the critical underlying epigenetic mechanisms of NaB is *via* the inhibition of HDAC, which leads to a reduction in the acetylation of NF-kB and P53 transcription factors (34, 35, 98). Moreover, HDAC inhibition is followed by PPARα upregulation, binding to p-p65 and H3K9Ac modifications on its promoter and eventually suppressing NF-kB-dependent signalling mechanisms (34). Additionally, NF-kB pathway suppression results in lowered IL levels (40). Cumulative evidence implies significant improvements in mRNA expression and protein levels of cytokines and chemokines after NaB administration (40). This evidence illustrates lowered pro-inflammatory cytokines, including IL-1β, IL-2 and IL-6 (23, 25, 32, 34, 35, 40, 99) as well as increased anti-inflammatory cytokines, including IL-4 and IL-10 (35, 40, 99). In contrast, one model of NASH-induced mice reported elevated levels of IL-1β expression after supplementation with oral NaB (600 mg/kg) for 6 weeks (31). Similarly, a more recent study showed permanent levels of ileum and elevated levels of IL-1β expression in the colon of NAFLD mice supplemented with NaB (5% diet) for 12 weeks (30). Moreover, serum levels of TNF-α were correlated with the severity of NAFLD (100). NaB administration has reduced the protein and gene expression levels of TNF-α in many NAFLD studies (23, 25, 32, 34, 35, 39, 40, 99).

Various pathways may play a role in the anti-inflammatory properties of NaB. Based on previous studies, TLR-4 signalling pathway induction enhances disease progression (39). TLR-4 induction is followed by an initial inflammatory response illustrated as cytokine production-inducing secondary TLR stimulation (101). Many studies have elaborated on the “TLR-4 inhibiting effect” of NaB (23, 25, 35, 39, 40).

In this context, NaB downregulates high mobility group box 1 mRNA expression and has an HDAC inhibitory effect that finally lowers TLR-4 levels (23, 102). As a component of “associated lipopolysaccharide mechanisms of NaB,” the downregulation of TLR-4/MyD88/NF-kB pathways inhibits NF-kB translocation (99). Eventually, the suppressed production of pro-inflammatory cytokines reduces hepatic and adipocyte pro-inflammatory cytokine gene expression, and enhanced anti-inflammatory cytokine gene expression is evident (99). NaB also suppresses the induction of TLR-4 and CD14 mRNA expression in the liver and TLR2 in the colon, improves cytokines and decreases Kupffer cell activation (35). Intragastric NaB (500 mg/kg/day) and/or *Clostridium butyricum* (5*10^7^ CFU/kg/day) for 6 weeks of treatment in db/db mice (with T2D-induced NAFLD) along with a Caco-2 cell culture supported the downregulation of the TLR-4/MyD88/NF-kB pathway, and the improvement was more significant in the group receiving *Clostridium butyricum* (25). It was also reported that 300 mg/kg/day of NaB in HFD-fed rats for 9 weeks suppressed obesity-induced inflammation in the adipocytes by inhibiting the NLRP3 inflammasome signalling pathway in the adipose tissue (34, 103). NaB significantly reduced colon inflammatory markers *via* F4/80 inhibition in mice and cellular models as well as improving gut inflammation (25).

Zhang et al. (32) also discovered hepatic changes in NAFLD-induced mice that had been gavaged with intragastric NaB (200 mg/kg/day). The results revealed the action of NaB on upregulating miR-150, which downregulates the expression of CXCR4, leading to protection against lobular inflammation and ballooning as detected by the NAFLD activity score (32). Another study showed the alleviation of macrophage infiltration parameters (MCP-1 and F4/80) as a predictor of inflammation-related tissue damage (40, 104). Additionally, Sun et al. (34) reported decreased levels of pro-inflammatory protein M1 macrophage marker F4/80 expression along with increased anti-inflammatory M2 macrophage marker CD206 after NaB gavage (300 mg/kg/day) for 7 weeks in NAFLD-induced rats.

Another favourable mechanism has been attributed to melatonin’s role in managing NAFLD (105). Jin et al. (39) and Baumann et al. (23) reported the efficacy of 600 mg/kg/day NaB for six and 5 weeks on increasing melatonin concentrations and related enzymes in a NAFLD mouse model. Melatonin reduces pro-inflammatory cytokines (106). The most recent *in vitro* study highlighted NaB’s multi-organ effects against inflammation (38). These findings suggest that NaB is a novel pharmacological agent for improving systemic and hepatic inflammation involved in NAFLD.

#### The effects of butyrate on oxidative-induced NAFLD

Beyond inflammation, liver steatosis leads to the formation of toxic lipid species that activate the vicious cycle of lipotoxicity and oxidative stress (107). Oxidative stress is an imbalance between pro-oxidant and anti-oxidant levels (108) that eventually affects organelles and results in cell death (109). The rapid oxidation of fatty acids in hepatic mitochondria leads to the production of high amounts of ROS, oxidative stress and NAFLD progression (110, 111). Oxidative stress can also accelerate fat deposition and inflammation (112). Despite studies highlighting the efficacy of anti-oxidant agents on NAFLD patients, a Cochrane meta-analysis has demonstrated that the effect of these drugs is still unconfirmed (113).

First, Jin et al. (31) investigated the effect of orally administering NaB (600 mg/kg/day) and its oxidative response in NASH-induced mice after 6 weeks. Results indicated that iNOS and 4-HNE protein adduct levels were normalised by the NaB supplementation (31). Further studies aligned with this result and demonstrated the indirect anti-oxidant effect of NaB on the liver (23, 39). This effect was mediated by increased intestinal levels of melatonin that exhibited anti-oxidant effects such as attenuating iNOS induction, ROS generation and hepatic lipid peroxidation (39). Interestingly, the same study showed that superoxide dismutase-1 (SOD1) activity was significantly lower in the group that received NaB (39). In contrast, other findings indicate the protective role of NaB against SOD reduction by elevating SOD2 mRNA expression levels and SOD enzyme activity per total liver tissue (32, 41). This controversy is related to different administration strategies of NaB (intraperitoneal vs. oral) (114). The underlying mechanism is related to the effect of NaB on regulating HDAC expression and binding levels to transcription factor fork-head box O3a (Foxo3a) (41). Foxo3a mediates the regulation of oxidative stress by increasing anti-oxidant enzyme expressions like SOD2 and catalase (115). Sun et al. (34) administrated NaB (300 mg/kg/day) *via* gavage on NALFD-induced rats for 9 weeks. Results illustrated an evident upregulated PPARs transcription factor expression, increased protein concentrations of cytochrome c oxidase subunit 1 (COX1), COX4 and mitochondrial complex III and V activity, indicating the favourable effects of NaB on mitochondrial function and fatty acid β-oxidation (34). Other animal studies also suggested the PPAR-dependent manner of NaB on lipid metabolism, fatty acid uptake and oxidation (35, 99). Honma et al. (41) refed Sprague–Dawley rats with a high-sucrose diet or a high-sucrose diet containing 5% NaB for 12 or 24 h. NaB significantly reduced the expression levels of genes involved in fatty acid synthesis and increased the genes involved in β-oxidation and modified mitochondria functions (including lowering FAS) as well as increasing Aco1 and carnitine palmitoyl transferase 1α (Cpt1α) expression (41). NaB has favourable effects on altering the enzymatic systems related to oxidative defence (116). As a biomarker for lipid peroxidation, a reduction in pancreatic and hepatic malondialdehyde was reported and attributed to oxidative stress (32, 117). Mitochondrial GSH regulates ROS; thus, GSH depletion is a critical factor in the progression of NASH (118). One study reported elevated Mn-SOD and catalase mRNA levels along with genes involved in the synthesis of GSH, including the NaB group; however, the activity of SOD, GSH levels and the GSSG/GSH ratio did not change significantly (41). Recent evidence suggests that NaB neither derives extra oxidative stress nor inserts defences against oxidative stress by increasing hepatic GSH (41).

Other marginal positive mechanisms of NaB that have been proposed based on *in vivo* and *in vitro* studies in NAFLD models may include (1) GLP-1 sensitising effects followed by enhanced hepatic fatty acid oxidation (33); (2) fibroblast growth factor-21 induction that subsequently stimulates hepatic fatty acid β-oxidation (35); (3) miR-150 upregulation followed by CXCR4 downregulation that eventually protects cells from oxidative stress (32); (4) nuclear factor erythroid 2-related factor 2 signalling alleviation, resulting in anti-oxidant genes expression, lipid peroxidation improvement and cell protection (117). Overall, NaB can be considered an anti-oxidative mediator in NAFLD.

## Knowledge gaps and future directions

Despite extensive investigative efforts, the underlying molecular mechanisms of NaB that affect NAFLD pathogenesis are still unclear. The mechanism of NaB action on NAFLD treatment has only been investigated in *in vivo* and *in vitro* studies, and there is a need for more clinical trials. More studies are needed to clarify these underlying mechanisms in HDAC inhibition and GPCR binding after NaB supplementation. Moreover, considering the potential role of *Clostridium butyricum* in previous studies, further investigation is needed to compare its effectiveness and NaB supplementation. Further studies are also necessary to examine the effect of NaB on melatonin-dependent molecular pathways and the eventual effect on NAFLD. Regardless of the commercial availability of NaB, the determination of appropriate formulation, suitable delivery systems and effective doses in NAFLD patients needs to be assessed in future trials.

## Conclusion

**FIGURE 2:**
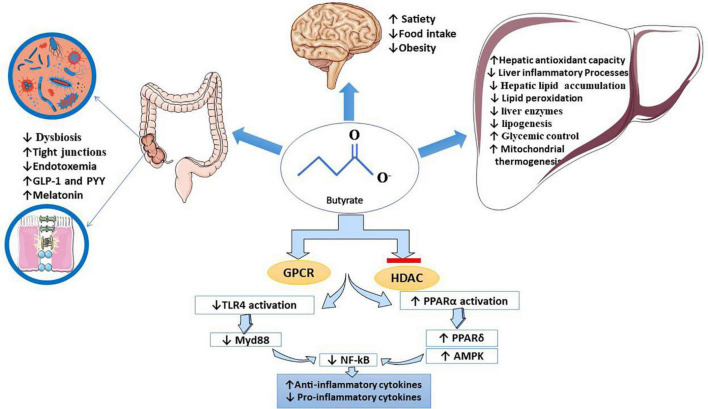
Butyrate’s beneficial effects on NAFLD. It has been proved that butyrate inhibits HDAC and binds to specific GPCRs, which hereby interferes with the expression of several genes. It downregulates TLR 4/MyD88/NF-kB pathways (inhibits NF-kB translocation) and upregulates PPAR-related pathways. Butyrate, mainly through these mechanisms, contributes to the management of NAFLD. Clinical manifestation of butyrate effects on the liver, gut, and brain pictured in this figure: (1) in the liver: butyrate improves liver enzymes, lipid metabolism, glycemic control, mitochondrial thermogenesis, oxidative status, hepatic lipid accumulation, and inflammatory markers, (2) in the gut: butyrate modifies dysbiosis and microbiota, reduces endotoxemia, increases intestinal integrity, increases anorexic hormones such as PYY and GLP-1 and modulates tight junction proteins, and (3) in the brain: as a positive effect of butyrate on managing obesity, it may suppress appetite and food intake. AMPK, adenosine monophosphate-activated protein kinase; GLP1, glucagon-like peptide-1; GPCR, G-protein coupled receptor; HDAC, histone deacetylase; MyD88, myeloid differentiation factor 88; NAFLD, non-alcoholic fatty liver disease; NF-kB, nuclear factor kappa-light-chain enhancer of activated B cells; PPAR, peroxisome proliferator-activated receptor; TJs, tight junctions; TLR-4, toll-like receptor-4.

This systematic review indicates that NaB contributes to NAFLD management by(a) improving intestinal homeostasis (modulating TJs and gut microbiota); (b) decreasing intrahepatic TG deposition by modulating hepatocytes lipid metabolism, which causes hepatic steatosis inhibition [(1) inhibition of hepatic lipogenesis; (2) increasing fatty acid oxidation; (3) upregulating miRNAs involved in pathways related to lipid metabolism; (4) stimulation of β-oxidation through the hepatic peroxisome proliferator-activated receptor α (PPARα) activation]; (c) reducing obesity by regulating energy metabolism (increasing energy expenditure, decreasing energy intake); (d) regulating blood glucose and lipid levels; (e) general and hepatic anti-inflammatory and anti-oxidative effects. All protective effects of NaB on different body organs are summarised in Figure 2. In conclusion, NaB’s beneficial effects on NAFLD and the associated risk factors may have potential applications in the prevention and management of NAFLD, but further research is needed to confirm suggestive findings.

## Data availability statement

The original contributions presented in this study are included in the article/supplementary material, further inquiries can be directed to the corresponding author.

## Author contributions

PA: concept development, study design, data collection, and manuscript drafting. SH: data interpretation, critical revising, and final approval. SA: study design and writing and revising the manuscript. FB: data collection and interpretation and writing the manuscript. HJ: writing and revising the manuscript. All authors contributed to the article and approved the submitted version.

## Abbreviations

ALT: alanine aminotransferase
AMPK: adenosine monophosphate-activated protein kinase
AST: aspartate aminotransferase
Caco-2: carcinoma colon-2
COX: cyclooxygenase
CD: cluster of differentiation
Cpt1α: carnitine palmitoyl transferase 1α
CXCL12: CXC chemokine
CXCR4: C-X-C chemokine receptor type 4
DNA: deoxyribonucleic acid
FAS: fatty acid synthase
FBA: N-(1-carbamoyl-2-phenyl-ethyl) butyramide
FBG: fasting blood glucose
FBS: fasting blood sugar
GLA: gut–liver axis
GLP1: glucagon-like peptide-1
GLP-1R: GLP-1 receptor
GPCR: G-protein-coupled receptor
GSH: glutathione
GSSG/GSH: oxidised glutathione/glutathione ratio
HDAC: histone deacetylase
HFD: high-fat diet
4-HNE: 4-hydroxynonenal
HOMA-IR: homeostatic model assessment of insulin resistance
IFN: interferon
IL: interleukin
iNOS: inducible nitric oxide synthase
INSIG: insulin-induced gene
IR: insulin resistance
ISI: insulin sensitivity index
LBP: lipopolysaccharide binding protein
LDL-C: low-density lipoprotein cholesterol
LPS: lipopolysaccharide
MAFLD: metabolic-associated fatty liver disease
MCP1: monocyte chemoattractant protein-1
MCS: methionine–choline-sufficient diet
MIM: metabolites of intestinal microflora
miR: microRNA
Mn-SOD: manganese superoxide dismutase
mRNA: messenger ribonucleic acid
NaB: sodium butyrate
NAFLD: non-alcoholic fatty liver disease
NAS: NAFLD activity score
NASH: non-alcoholic steatohepatitis
NF-kB: nuclear factor kappa-light-chain-enhancer of activated B cells
PPAR: peroxisome proliferator-activated receptor
SCFAs: short-chain fatty acids
ROS: radical oxygen species
SOD1: superoxide dismutase-1
SREBP: sterol regulatory element-binding protein
TC: total cholesterol
TG: triglyceride
TJs: tight junctions
TLR: toll-like receptor
TNF-α: tumour necrosis factor α
WSD: western-style diet.

## References

[1] Perumpail BJ Khan MA Yoo ER Cholankeril G Kim D Ahmed A. Clinical epidemiology and disease burden of nonalcoholic fatty liver disease. *World J Gastroenterol.* (2017) 23:8263. 10.3748/wjg.v23.i47.8263 29307986 PMC5743497

[2] Chalasani N Younossi Z Lavine JE Diehl AM Brunt EM Cusi K The diagnosis and management of non-alcoholic fatty liver disease: practice guideline by the American Gastroenterological Association, American Association for the Study of Liver Diseases, and American College of Gastroenterology. *Gastroenterology.* (2012) 142:1592–609. 10.1053/j.gastro.2012.04.001 22656328

[3] Ramai D Tai W Rivera M Facciorusso A Tartaglia N Pacilli M Natural progression of non-alcoholic steatohepatitis to hepatocellular carcinoma. *Biomedicines.* (2021) 9:184. 10.3390/biomedicines9020184 33673113 PMC7918599

[4] Huang DQ El-Serag HB Loomba R. Global epidemiology of NAFLD-related HCC: trends, predictions, risk factors and prevention. *Nat Rev Gastroenterol Hepatol.* (2021) 18:223–38. 10.1038/s41575-020-00381-6 33349658 PMC8016738

[5] Pafili K Roden M. Nonalcoholic fatty liver disease (NAFLD) from pathogenesis to treatment concepts in humans. *Mol Metab.* (2021) 50:101122. 10.1016/j.molmet.2020.101122 33220492 PMC8324683

[6] Friedman SL Neuschwander-Tetri BA Rinella M Sanyal AJ. Mechanisms of NAFLD development and therapeutic strategies. *Nat Med.* (2018) 24:908–22. 10.1038/s41591-018-0104-9 29967350 PMC6553468

[7] Yki-Järvinen H. Non-alcoholic fatty liver disease as a cause and a consequence of metabolic syndrome. *Lancet Diabetes Endocrinol.* (2014) 2:901–10. 10.1016/S2213-8587(14)70032-4 24731669

[8] Koliaki C Szendroedi J Kaul K Jelenik T Nowotny P Jankowiak F Adaptation of hepatic mitochondrial function in humans with non-alcoholic fatty liver is lost in steatohepatitis. *Cell Metab.* (2015) 21:739–46. 10.1016/j.cmet.2015.04.004 25955209

[9] Petersen MC Madiraju AK Gassaway BM Marcel M Nasiri AR Butrico G Insulin receptor Thr 1160 phosphorylation mediates lipid-induced hepatic insulin resistance. *J Clin Invest.* (2016) 126:4361–71. 10.1172/JCI86013 27760050 PMC5096902

[10] Marchisello S Di Pino A Scicali R Urbano F Piro S Purrello F Pathophysiological, molecular and therapeutic issues of nonalcoholic fatty liver disease: an overview. *Int J Mol Sci.* (2019) 20:1948. 10.3390/ijms20081948 31010049 PMC6514656

[11] Lonardo A Nascimbeni F Targher G Bernardi M Bonino F Bugianesi E AISF position paper on nonalcoholic fatty liver disease (NAFLD): updates and future directions. *Digest Liver Dis.* (2017) 49:471–83. 10.1016/j.dld.2017.01.147 28215516

[12] Trauner M Arrese M Wagner M. Fatty liver and lipotoxicity. *Biochim Biophys Acta Mol Cell Biol Lipids.* (2010) 1801:299–310. 10.1016/j.bbalip.2009.10.007 19857603

[13] Masarone M Rosato V Dallio M Gravina AG Aglitti A Loguercio C Role of oxidative stress in pathophysiology of nonalcoholic fatty liver disease. *Oxid Med Cell Longev.* (2018) 2018:9547613. 10.1155/2018/9547613 29991976 PMC6016172

[14] Srinivas AN Suresh D Santhekadur PK Suvarna D Kumar DP. Extracellular vesicles as inflammatory drivers in NAFLD. *Front Immunol.* (2021) 11:3745. 10.3389/fimmu.2020.627424 33603757 PMC7884478

[15] Albillos A De Gottardi A Rescigno M. The gut-liver axis in liver disease: pathophysiological basis for therapy. *J Hepatol.* (2020) 72:558–77. 10.1016/j.jhep.2019.10.003 31622696

[16] Jasirwan COM Lesmana CRA Hasan I Sulaiman AS Gani RA. The role of gut microbiota in non-alcoholic fatty liver disease: pathways of mechanisms. *Biosci Microbiota Food Health.* (2019) 38:81–8. 10.12938/bmfh.18-032 31384519 PMC6663510

[17] Grabherr F Grander C Effenberger M Adolph TE Tilg H. Gut dysfunction and non-alcoholic fatty liver disease. *Front Endocrinol.* (2019) 10:611. 10.3389/fendo.2019.00611 31555219 PMC6742694

[18] Ji Y Yin Y Li Z Zhang W. Gut microbiota-derived components and metabolites in the progression of non-alcoholic fatty liver disease (NAFLD). *Nutrients.* (2019) 11:1712. 10.3390/nu11081712 31349604 PMC6724003

[19] Da Zhou J-GF. Microbial metabolites in non-alcoholic fatty liver disease. *World J Gastroenterol.* (2019) 25:2019–28. 10.3748/wjg.v25.i17.2019 31114130 PMC6506577

[20] Saltzman ET Palacios T Thomsen M Vitetta L. Intestinal microbiome shifts, dysbiosis, inflammation, and non-alcoholic fatty liver disease. *Front Microbiol.* (2018) 9:61. 10.3389/fmicb.2018.00061 29441049 PMC5797576

[21] EASL, EASD, EASO. EASL-EASD-EASO clinical practice guidelines for the management of non-alcoholic fatty liver disease. *J Hepatol.* (2016) 64:1388–402. 10.1016/j.jhep.2015.11.004 27062661

[22] Arefhosseini S Tutunchi H Golzar S Mahboob S Pouretedal Z Ebrahimi-Mameghani M. The effect of hydroxy citric acid supplementation with calorie-restricted diet on metabolic, atherogenic and inflammatory biomarkers in women with non-alcoholic fatty liver disease: a randomized controlled clinical trial. *Food Funct.* (2022) 13:5124–34. 10.1039/D1FO03685H 35416190

[23] Baumann A Jin CJ Brandt A Sellmann C Nier A Burkard M Oral supplementation of sodium butyrate attenuates the progression of non-alcoholic steatohepatitis. *Nutrients.* (2020) 12:951. 10.3390/nu12040951 32235497 PMC7231312

[24] Bourassa MW Alim I Bultman SJ Ratan RR. Butyrate, neuroepigenetics and the gut microbiome: can a high fiber diet improve brain health? *Neurosci Lett.* (2016) 625:56–63. 10.1016/j.neulet.2016.02.009 26868600 PMC4903954

[25] Yang T Yang H Heng C Wang H Chen S Hu Y Amelioration of non-alcoholic fatty liver disease by sodium butyrate is linked to the modulation of intestinal tight junctions in db/db mice. *Food Funct.* (2020) 11:10675–89. 10.1039/D0FO01954B 33216087

[26] Canani RB Di Costanzo M Leone L Pedata M Meli R Calignano A. Potential beneficial effects of butyrate in intestinal and extraintestinal diseases. *World J Gastroenterol.* (2011) 17:1519. 10.3748/wjg.v17.i12.1519 21472114 PMC3070119

[27] Bridgeman SC Northrop W Melton PE Ellison GC Newsholme P Mamotte CD. Butyrate generated by gut microbiota and its therapeutic role in metabolic syndrome. *Pharmacol Res.* (2020) 160:105174. 10.1016/j.phrs.2020.105174 32860943

[28] de Clercq NC Groen AK Romijn JA Nieuwdorp M. Gut microbiota in obesity and undernutrition. *Adv Nutr.* (2016) 7:1080–9. 10.3945/an.116.012914 28140325 PMC5105041

[29] Tan J McKenzie C Potamitis M Thorburn AN Mackay CR Macia L. The role of short-chain fatty acids in health and disease. *Adv Immunol.* (2014) 121:91–119. 10.1016/B978-0-12-800100-4.00003-9 24388214

[30] Beisner J Filipe Rosa L Kaden-Volynets V Stolzer I Günther C Bischoff SC. Prebiotic inulin and sodium butyrate attenuate obesity-induced intestinal barrier dysfunction by induction of antimicrobial peptides. *Front Immunol.* (2021) 12:678360. 10.3389/fimmu.2021.678360 34177920 PMC8226265

[31] Jin CJ Sellmann C Engstler AJ Ziegenhardt D Bergheim I. Supplementation of sodium butyrate protects mice from the development of non-alcoholic steatohepatitis (NASH). *Br J Nutr.* (2015) 114:1745–55. 10.1017/S0007114515003621 26450277

[32] Zhang N Qu Y Qin B. Sodium butyrate ameliorates non-alcoholic fatty liver disease by upregulating miR-150 to suppress CXCR4 expression. *Clin Exp Pharmacol Physiol.* (2021) 48:1125–36. 10.1111/1440-1681.13497 33721354

[33] Zhao ZH Wang ZX Zhou D Han Y Ma F Hu Z Sodium butyrate supplementation inhibits hepatic steatosis by stimulating liver kinase B1 and insulin-induced gene. *Cell Mol Gastroenterol Hepatol.* (2021) 12:857–71. 10.1016/j.jcmgh.2021.05.006 33989817 PMC8346675

[34] Sun B Jia Y Hong J Sun Q Gao S Hu Y Sodium butyrate ameliorates high-fat-diet-induced non-alcoholic fatty liver disease through peroxisome proliferator-activated receptor α-mediated activation of β oxidation and suppression of inflammation. *J Agric Food Chem.* (2018) 66:7633–42. 10.1021/acs.jafc.8b01189 29961332

[35] Ye J Lv L Wu W Li Y Shi D Fang D Butyrate protects mice against methionine-choline-deficient diet-induced non-alcoholic steatohepatitis by improving gut barrier function, attenuating inflammation and reducing endotoxin levels. *Front Microbiol.* (2018) 9:1967. 10.3389/fmicb.2018.01967 30186272 PMC6111843

[36] Zhou D Chen Y-W Zhao Z-H Yang R-X Xin F-Z Liu X-L Sodium butyrate reduces high-fat diet-induced non-alcoholic steatohepatitis through upregulation of hepatic GLP-1R expression. *Exp Mol Med.* (2018) 50:1–12. 10.1038/s12276-018-0183-1 30510243 PMC6277380

[37] Zhou D Pan Q Xin FZ Zhang RN He CX Chen GY Sodium butyrate attenuates high-fat diet-induced steatohepatitis in mice by improving gut microbiota and gastrointestinal barrier. *World J Gastroenterol.* (2017) 23:60–75. 10.3748/wjg.v23.i1.60 28104981 PMC5221287

[38] Prins GH Rios-Morales M Gerding A Reijngoud DJ Olinga P Bakker BM. The effects of butyrate on induced metabolic-associated fatty liver disease in precision-cut liver slices. *Nutrients.* (2021) 13:4203. 10.3390/nu13124203 34959755 PMC8703944

[39] Jin CJ Engstler AJ Sellmann C Ziegenhardt D Landmann M Kanuri G Sodium butyrate protects mice from the development of the early signs of non-alcoholic fatty liver disease: role of melatonin and lipid peroxidation. *Br J Nutr.* (2016) 116:1682–93. 10.1017/S0007114516004025 27876107

[40] Mattace Raso G Simeoli R Russo R Iacono A Santoro A Paciello O Effects of sodium butyrate and its synthetic amide derivative on liver inflammation and glucose tolerance in an animal model of steatosis induced by high fat diet. *PLoS One.* (2013) 8:e68626. 10.1371/journal.pone.0068626 23861927 PMC3702592

[41] Honma K Oshima K Takami S Goda T. Regulation of hepatic genes related to lipid metabolism and antioxidant enzymes by sodium butyrate supplementation. *Metabol Open.* (2020) 7:100043. 10.1016/j.metop.2020.100043 32812944 PMC7424775

[42] Chen W Zhang S Wu J Ye T Wang S Wang P Butyrate-producing bacteria and the gut-heart axis in atherosclerosis. *Clin Chim Acta.* (2020) 507:236–41. 10.1016/j.cca.2020.04.037 32376324

[43] Liu H Wang J He T Becker S Zhang G Li D Butyrate: a double-edged sword for health? *Adv Nutr.* (2018) 9:21–9. 10.1093/advances/nmx009 29438462 PMC6333934

[44] Louis P Flint HJ. Formation of propionate and butyrate by the human colonic microbiota. *Environ Microbiol.* (2017) 19:29–41. 10.1111/1462-2920.13589 27928878

[45] van der Beek CM Bloemen JG van den Broek MA Lenaerts K Venema K Buurman WA Hepatic uptake of rectally administered butyrate prevents an increase in systemic butyrate concentrations in humans. *J Nutr.* (2015) 145:2019–24. 10.3945/jn.115.211193 26156796

[46] Silva YP Bernardi A Frozza RL. The role of short-chain fatty acids from gut microbiota in gut-brain communication. *Front Endocrinol.* (2020) 11:25. 10.3389/fendo.2020.00025 32082260 PMC7005631

[47] Leonel AJ Alvarez-Leite JI. Butyrate: implications for intestinal function. *Curr Opin Clin Nutr Metab Care.* (2012) 15:474–9. 10.1097/MCO.0b013e32835665fa 22797568

[48] Fianchi F Liguori A Gasbarrini A Grieco A Miele L. Nonalcoholic fatty liver disease (Nafld) as model of gut–liver axis interaction: from pathophysiology to potential target of treatment for personalized therapy. *Int J Mol Sci.* (2021) 22:6485. 10.3390/ijms22126485 34204274 PMC8233936

[49] Wang J Tang H Zhang C Zhao Y Derrien M Rocher E Modulation of gut microbiota during probiotic-mediated attenuation of metabolic syndrome in high fat diet-fed mice. *ISME J.* (2015) 9:1–15. 10.1038/ismej.2014.99 24936764 PMC4274436

[50] Da Silva HE Teterina A Comelli EM Taibi A Arendt BM Fischer SE Nonalcoholic fatty liver disease is associated with dysbiosis independent of body mass index and insulin resistance. *Sci Rep.* (2018) 8:1–12. 10.1038/s41598-018-19753-9 29362454 PMC5780381

[51] Endo H Niioka M Kobayashi N Tanaka M Watanabe T. Butyrate-producing probiotics reduce nonalcoholic fatty liver disease progression in rats: new insight into the probiotics for the gut-liver axis. *PLoS One.* (2013) 8:e63388. 10.1371/journal.pone.0063388 23696823 PMC3656030

[52] Wang H-B Wang P-Y Wang X Wan Y-L Liu Y-C. Butyrate enhances intestinal epithelial barrier function via up-regulation of tight junction protein claudin-1 transcription. *Digest Dis Sci.* (2012) 57:3126–35. 10.1007/s10620-012-2259-4 22684624

[53] Ma X Fan P Li L Qiao S Zhang G Li D. Butyrate promotes the recovering of intestinal wound healing through its positive effect on the tight junctions. *J Anim Sci.* (2012) 90(Suppl. 4):266–8. 10.2527/jas.50965 23365351

[54] Bauer H Zweimueller-Mayer J Steinbacher P Lametschwandtner A Bauer H-C. The dual role of zonula occludens (ZO) proteins. *J Biomed Biotechnol.* (2010) 2010:402593. 10.1155/2010/402593 20224657 PMC2836178

[55] Etienne-Mesmin L Vijay-Kumar M Gewirtz AT Chassaing B. Hepatocyte toll-like receptor 5 promotes bacterial clearance and protects mice against high-fat diet–induced liver disease. *Cell Mol Gastroenterol Hepatol.* (2016) 2:584–604. 10.1016/j.jcmgh.2016.04.007 28090564 PMC5042709

[56] Vancamelbeke M Vermeire S. The intestinal barrier: a fundamental role in health and disease. *Expert Rev Gastroenterol Hepatol.* (2017) 11:821–34. 10.1080/17474124.2017.1343143 28650209 PMC6104804

[57] Ben-Shlomo S Zvibel I Shnell M Shlomai A Chepurko E Halpern Z Glucagon-like peptide-1 reduces hepatic lipogenesis via activation of AMP-activated protein kinase. *J Hepatol.* (2011) 54:1214–23. 10.1016/j.jhep.2010.09.032 21145820

[58] Trevaskis JL Griffin PS Wittmer C Neuschwander-Tetri BA Brunt EM Dolman CS Glucagon-like peptide-1 receptor agonism improves metabolic, biochemical, and histopathological indices of nonalcoholic steatohepatitis in mice. *Am J Physiol Gastrointest Liver Physiol.* (2012) 302:G762–72. 10.1152/ajpgi.00476.2011 22268099

[59] Noureldein MH Bitar S Youssef N Azar S Eid AA. Butyrate modulates diabetes-linked gut dysbiosis: epigenetic and mechanistic modifications. *J Mol Endocrinol.* (2020) 64:29–42. 10.1530/JME-19-0132 31770101

[60] Pouwels S Sakran N Graham Y Leal A Pintar T Yang W Non-alcoholic fatty liver disease (NAFLD): a review of pathophysiology, clinical management and effects of weight loss. *BMC Endocrine Disord.* (2022) 22:63. 10.1186/s12902-022-00980-1 35287643 PMC8919523

[61] Alkhouri N Lawitz E Noureddin M DeFronzo R Shulman GI. GS-0976 (Firsocostat): an investigational liver-directed acetyl-CoA carboxylase (ACC) inhibitor for the treatment of non-alcoholic steatohepatitis (NASH). *Expert Opin Invest Drugs.* (2020) 29:135–41. 10.1080/13543784.2020.1668374 31519114 PMC7063378

[62] Smith GI Shankaran M Yoshino M Schweitzer GG Chondronikola M Beals JW Insulin resistance drives hepatic de novo lipogenesis in nonalcoholic fatty liver disease. *J Clin Invest.* (2020) 130:1453–60. 10.1172/JCI134165 31805015 PMC7269561

[63] Engelking LJ Kuriyama H Hammer RE Horton JD Brown MS Goldstein JL Overexpression of insig-1 in the livers of transgenic mice inhibits SREBP processing and reduces insulin-stimulated lipogenesis. *J Clin Invest.* (2004) 113:1168–75. 10.1172/JCI20978 15085196 PMC385408

[64] Li Y Xu S Mihaylova MM Zheng B Hou X Jiang B AMPK phosphorylates and inhibits SREBP activity to attenuate hepatic steatosis and atherosclerosis in diet-induced insulin-resistant mice. *Cell Metab.* (2011) 13:376–88. 10.1016/j.cmet.2011.03.009 21459323 PMC3086578

[65] Lyseng-Williamson KA. Correction to: glucagon-like peptide-1 receptor agonists in type 2 diabetes: their use and differential features. *Clin Drug Invest.* (2019) 39:1019. 10.1007/s40261-019-00852-y 31512163 PMC6765691

[66] Ceccarelli S Panera N Gnani D Nobili V. Dual role of microRNAs in NAFLD. *Int J Mol Sci.* (2013) 14:8437–55. 10.3390/ijms14048437 23594995 PMC3645753

[67] Karin N. The multiple faces of CXCL12 (SDF-1α) in the regulation of immunity during health and disease. *J Leukocyte Biol.* (2010) 88:463–73. 10.1189/jlb.0909602 20501749

[68] Paradies G Paradies V Ruggiero FM Petrosillo G. Oxidative stress, cardiolipin and mitochondrial dysfunction in nonalcoholic fatty liver disease. *World J Gastroenterol.* (2014) 20:14205. 10.3748/wjg.v20.i39.14205 25339807 PMC4202349

[69] Montagner A Polizzi A Fouché E Ducheix S Lippi Y Lasserre F Liver PPARα is crucial for whole-body fatty acid homeostasis and is protective against NAFLD. *Gut.* (2016) 65:1202–14. 10.1136/gutjnl-2015-310798 26838599 PMC4941147

[70] Ajmera V Loomba R. Imaging biomarkers of NAFLD, NASH, and fibrosis. *Mol Metab.* (2021) 50:101167. 10.1016/j.molmet.2021.101167 33460786 PMC8324681

[71] Zhang L Liu C Jiang Q Yin Y. Butyrate in energy metabolism: there is still more to learn. *Trends Endocrinol Metab.* (2021) 32:159–69. 10.1016/j.tem.2020.12.003 33461886

[72] Lin HV Frassetto A Kowalik EJ Jr Nawrocki AR Lu MM Kosinski JR Butyrate and propionate protect against diet-induced obesity and regulate gut hormones via free fatty acid receptor 3-independent mechanisms. *PLoS One.* (2012) 7:e35240. 10.1371/journal.pone.0035240 22506074 PMC3323649

[73] Amiri P Hosseini SA Ghaffari S Tutunchi H Ghaffari S Mosharkesh E Role of butyrate, a gut microbiota derived metabolite, in cardiovascular diseases: a comprehensive narrative review. *Front Pharmacol.* (2022) 12:837509. 10.3389/fphar.2021.837509 35185553 PMC8847574

[74] Bello-Chavolla OY Kuri-García A Ríos-Ríos M Vargas-Vázquez A Cortés-Arroyo JE Tapia-González G Familial combined hyperlipidemia: current knowledge, perspectives, and controversies. *Rev Invest Clin.* (2018) 70:224–36. 10.24875/RIC.18002575 30307446

[75] Hara H Haga S Aoyama Y Kiriyama S. Short-chain fatty acids suppress cholesterol synthesis in rat liver and intestine. *J Nutr.* (1999) 129:942–8. 10.1093/jn/129.5.942 10222383

[76] Coppola S Avagliano C Calignano A Berni Canani R. The protective role of butyrate against obesity and obesity-related diseases. *Molecules.* (2021) 26:682. 10.3390/molecules26030682 33525625 PMC7865491

[77] Alvaro A Solà R Rosales R Ribalta J Anguera A Masana L Gene expression analysis of a human enterocyte cell line reveals downregulation of cholesterol biosynthesis in response to short-chain fatty acids. *IUBMB Life.* (2008) 60:757–64. 10.1002/iub.110 18642346

[78] Luo J Yang H Song BL. Mechanisms and regulation of cholesterol homeostasis. *Nat Rev Mol Cell Biol.* (2020) 21:225–45. 10.1038/s41580-019-0190-7 31848472

[79] Marcil V Delvin E Seidman E Poitras L Zoltowska M Garofalo C Modulation of lipid synthesis, apolipoprotein biogenesis, and lipoprotein assembly by butyrate. *Am J Physiol Gastrointest Liver Physiol.* (2002) 283:G340–6. 10.1152/ajpgi.00440.2001 12121881

[80] Xiao Y Guo Z Li Z Ling H Song C. Role and mechanism of action of butyrate in atherosclerotic diseases: a review. *J Appl Microbiol.* (2021) 131:543–52. 10.1111/jam.14906 33098194

[81] Zhang L Du J Yano N Wang H Zhao YT Dubielecka PM Sodium butyrate protects -against high fat diet-induced cardiac dysfunction and metabolic disorders in type II diabetic mice. *J Cell Biochem.* (2017) 118:2395–408. 10.1002/jcb.25902 28109123 PMC5462877

[82] Chistiakov DA Bobryshev YV Orekhov AN. Macrophage-mediated cholesterol handling in atherosclerosis. *J Cell Mol Med.* (2016) 20:17–28. 10.1111/jcmm.12689 26493158 PMC4717859

[83] Viswanathan V Kadiri M Medimpudi S Kumpatla S. Association of non-alcoholic fatty liver disease with diabetic microvascular and macrovascular complications in South Indian diabetic subjects. *Int J Diabetes Dev Count.* (2010) 30:3022–7. 10.4103/0973-3930.70861

[84] Javeed N Matveyenko AV. Circadian etiology of type 2 diabetes mellitus. *Physiology.* (2018) 33:138–50. 10.1152/physiol.00003.2018 29412061 PMC5899235

[85] Candon S Perez-Arroyo A Marquet C Valette F Foray A-P Pelletier B Antibiotics in early life alter the gut microbiome and increase disease incidence in a spontaneous mouse model of autoimmune insulin-dependent diabetes. *PLoS One.* (2015) 10:e0125448. 10.1371/journal.pone.0125448 25970503 PMC4430542

[86] Hu Y Liu J Yuan Y Chen J Cheng S Wang H Sodium butyrate mitigates type 2 diabetes by inhibiting PERK-CHOP pathway of endoplasmic reticulum stress. *Environ Toxicol Pharmacol.* (2018) 64:112–21. 10.1016/j.etap.2018.09.002 30342372

[87] Khan S Jena G. Sodium butyrate reduces insulin-resistance, fat accumulation and dyslipidemia in type-2 diabetic rat: a comparative study with metformin. *Chem Biol Interact.* (2016) 254:124–34. 10.1016/j.cbi.2016.06.007 27270450

[88] Oiso H Furukawa N Suefuji M Shimoda S Ito A Furumai R The role of class I histone deacetylase (HDAC) on gluconeogenesis in liver. *Biochem Biophys Res Commun.* (2011) 404:166–72. 10.1016/j.bbrc.2010.11.086 21108932

[89] Yadav H Lee J-H Lloyd J Walter P Rane SG. Beneficial metabolic effects of a probiotic via butyrate-induced GLP-1 hormone secretion. *J Biol Chem.* (2013) 288:25088–97. 10.1074/jbc.M113.452516 23836895 PMC3757173

[90] Christiansen CB Gabe MBN Svendsen B Dragsted LO Rosenkilde MM Holst JJ. The impact of short-chain fatty acids on GLP-1 and PYY secretion from the isolated perfused rat colon. *Am J Physiol Gastrointest Liver Physiol.* (2018) 315:G53–65. 10.1152/ajpgi.00346.2017 29494208

[91] Pan X Wen SW Kaminga AC Liu A. Gut metabolites and inflammation factors in non-alcoholic fatty liver disease: a systematic review and meta-analysis. *Sci Rep.* (2020) 10:1–11. 10.1038/s41598-020-65051-8 32483129 PMC7264254

[92] Dludla PV Nkambule BB Mazibuko-Mbeje SE Nyambuya TM Marcheggiani F Cirilli I N-acetyl cysteine targets hepatic lipid accumulation to curb oxidative stress and inflammation in NAFLD: a comprehensive analysis of the literature. *Antioxidants.* (2020) 9:1283. 10.3390/antiox9121283 33339155 PMC7765616

[93] Mann JP Raponi M Nobili V. Clinical implications of understanding the association between oxidative stress and pediatric NAFLD. *Expert Rev Gastroenterol Hepatol.* (2017) 11:371–82. 10.1080/17474124.2017.1291340 28162008

[94] Hammerich L Tacke F. Interleukins in chronic liver disease: lessons learned from experimental mouse models. *Clin Exp Gastroenterol.* (2014) 7:297. 10.2147/CEG.S43737 25214799 PMC4158890

[95] Adams LA Anstee QM Tilg H Targher G. Non-alcoholic fatty liver disease and its relationship with cardiovascular disease and other extrahepatic diseases. *Gut.* (2017) 66:1138–53. 10.1136/gutjnl-2017-313884 28314735

[96] Kanika G Khan S Jena G. Sodium butyrate ameliorates L-arginine-induced pancreatitis and associated fibrosis in Wistar rat: role of inflammation and nitrosative stress. *J Biochem Mol Toxicol.* (2015) 29:349–59. 10.1002/jbt.21698 25774002

[97] Ohira H Fujioka Y Katagiri C Mamoto R Aoyama-Ishikawa M Amako K Butyrate attenuates inflammation and lipolysis generated by the interaction of adipocytes and macrophages. *J Atheroscler Thromb.* (2013) 20:425–42. 10.5551/jat.15065 23470566

[98] Glozak MA Sengupta N Zhang X Seto E. Acetylation and deacetylation of non-histone proteins. *Gene.* (2005) 363:15–23. 10.1016/j.gene.2005.09.010 16289629

[99] Da Zhou QP Xin F-Z Zhang R-N He C-X Chen G-Y Liu C Sodium butyrate attenuates high-fat diet-induced steatohepatitis in mice by improving gut microbiota and gastrointestinal barrier. *World J Gastroenterol.* (2017) 23:60. 28104981 10.3748/wjg.v23.i1.60 PMC5221287

[100] Stojsavljević S Palčić MG Jukić LV Duvnjak LS Duvnjak M. Adipokines and proinflammatory cytokines, the key mediators in the pathogenesis of nonalcoholic fatty liver disease. *World J Gastroenterol.* (2014) 20:18070. 10.3748/wjg.v20.i48.18070 25561778 PMC4277948

[101] Szabo G Billiar TR Machida K Crispe IN Seki E. Toll-like receptor signaling in liver diseases. *Hindawi.* (2010) 2010:971270. 10.1155/2010/971270 21789039 PMC3123973

[102] Li L Chen L Hu L Liu Y Sun HY Tang J Nuclear factor high-mobility group box1 mediating the activation of Toll-like receptor 4 signaling in hepatocytes in the early stage of nonalcoholic fatty liver disease in mice. *Hepatology.* (2011) 54:1620–30. 10.1002/hep.24552 21809356

[103] Wang X He G Peng Y Zhong W Wang Y Zhang B. Sodium butyrate alleviates adipocyte inflammation by inhibiting NLRP3 pathway. *Sci Rep.* (2015) 5:1–10. 10.1038/srep12676 26234821 PMC4522654

[104] Kanda H Tateya S Tamori Y Kotani K Hiasa K-I Kitazawa R MCP-1 contributes to macrophage infiltration into adipose tissue, insulin resistance, and hepatic steatosis in obesity. *J Clin Invest.* (2006) 116:1494–505. 10.1172/JCI26498 16691291 PMC1459069

[105] Celinski K Konturek PC Slomka M Cichoz-Lach H Brzozowski T Konturek SJ Effects of treatment with melatonin and tryptophan on liver enzymes, parameters of fat metabolism and plasma levels of cytokines in patients with non-alcoholic fatty liver disease–14 months follow up. *J Physiol Pharmacol.* (2014) 65:75–82. 24622832

[106] Hatzis G Ziakas P Kavantzas N Triantafyllou A Sigalas P Andreadou I Melatonin attenuates high fat diet-induced fatty liver disease in rats. *World J Hepatol.* (2013) 5:160. 10.4254/wjh.v5.i4.160 23671720 PMC3648647

[107] Marra F Svegliati-Baroni G. Lipotoxicity and the gut-liver axis in NASH pathogenesis. *J Hepatol.* (2018) 68:280–95. 10.1016/j.jhep.2017.11.014 29154964

[108] Rives C Fougerat A Ellero-Simatos S Loiseau N Guillou H Gamet-Payrastre L Oxidative stress in NAFLD: role of nutrients and food contaminants. *Biomolecules.* (2020) 10:1702. 10.3390/biom10121702 33371482 PMC7767499

[109] Chen Z Tian R She Z Cai J Li H. Role of oxidative stress in the pathogenesis of nonalcoholic fatty liver disease. *Free Radic Biol Med.* (2020) 152:116–41. 10.1016/j.freeradbiomed.2020.02.025 32156524

[110] Fernández-Sánchez A Madrigal-Santillán E Bautista M Esquivel-Soto J Morales-González Á Esquivel-Chirino C Inflammation, oxidative stress, and obesity. *Int J Mol Sci.* (2011) 12:3117–32. 10.3390/ijms12053117 21686173 PMC3116179

[111] Tariq Z Green CJ Hodson L. Are oxidative stress mechanisms the common denominator in the progression from hepatic steatosis towards non-alcoholic steatohepatitis (NASH)? *Liver Int.* (2014) 34:e180–90. 10.1111/liv.12523 24621397

[112] Kobori M Akimoto Y Takahashi Y Kimura T. Combined effect of quercetin and fish oil on oxidative stress in the liver of mice fed a Western-style diet. *J Agric Food Chem.* (2020) 68:13267–75. 10.1021/acs.jafc.0c02984 32786869

[113] Lombardi R Onali S Thorburn D Davidson BR Gurusamy KS Tsochatzis E. Pharmacological interventions for non-alcohol related fatty liver disease (NAFLD). *Cochrane Database Syst Rev.* (2017) 3:CD011640. 10.1002/14651858.CD011640.pub2 28358980 PMC6464620

[114] Hu X Zhang K Xu C Chen Z Jiang H. Anti-inflammatory effect of sodium butyrate preconditioning during myocardial ischemia/reperfusion. *Exp Therap Med.* (2014) 8:229–32. 10.3892/etm.2014.1726 24944626 PMC4061237

[115] Huang H Tindall DJ. Dynamic FoxO transcription factors. *J Cell Sci.* (2007) 120:2479–87. 10.1242/jcs.001222 17646672

[116] Russo I Luciani A De Cicco P Troncone E Ciacci C. Butyrate attenuates lipopolysaccharide-induced inflammation in intestinal cells and Crohn’s mucosa through modulation of antioxidant defense machinery. *PLoS One.* (2012) 7:e32841. 10.1371/journal.pone.0032841 22412931 PMC3295784

[117] Adeyanju OA Badejogbin OC Areola DE Olaniyi KS Dibia C Soetan OA Sodium butyrate arrests pancreato-hepatic synchronous uric acid and lipid dysmetabolism in high fat diet fed Wistar rats. *Biomed Pharmacother.* (2021) 133:110994. 10.1016/j.biopha.2020.110994 33197764

[118] Mantena SK King AL Andringa KK Eccleston HB Bailey SM. Mitochondrial dysfunction and oxidative stress in the pathogenesis of alcohol-and obesity-induced fatty liver diseases. *Free Radic Biol Med.* (2008) 44:1259–72. 10.1016/j.freeradbiomed.2007.12.029 18242193 PMC2323912

